# Depletion of 14-3-3γ reduces the surface expression of Transient Receptor Potential Melastatin 4b (TRPM4b) Channels and attenuates TRPM4b-mediated glutamate-induced neuronal cell death

**DOI:** 10.1186/s13041-014-0052-3

**Published:** 2014-07-22

**Authors:** Chang-Hoon Cho, Eunju Kim, Young-Sun Lee, Oleg Yarishkin, Jae Cheal Yoo, Jae-Yong Park, Seong-Geun Hong, Eun Mi Hwang

**Affiliations:** 1 WCI Center for functional connectomics, Korea Institute of Science and Technology (KIST), Seoul 136-791, Republic of Korea; 2 College of Life Sciences and Biotechnology, Korea University, Anam-ro 145, Seongbuk-gu, Seoul, Republic of Korea; 3 Department of Physiology, Institute of Health Science and Medical Research Center for Neural Dysfunction, Gyeongsang National University School of Medicine, Jinju 660-51, Republic of Korea; 4 Neuroscience program, University of Science and Technology (UST), Daejeon 305-350, Republic of Korea

**Keywords:** TRPM4b, 14-3-3, Non-selective cation channels, 9-phenanthrol, HT-22, Protein-protein interaction, Surface expression, Hippocampal neurons, Calcium activated cation channels, MTT assay

## Abstract

**Background:**

TRPM4 channels are Ca^2+^-activated nonselective cation channels which are deeply involved in physiological and pathological conditions. However, their trafficking mechanism and binding partners are still elusive.

**Results:**

We have found the 14-3-3γ as a binding partner for TRPM4b using its N-terminal fragment from the yeast-two hybrid screening. Ser88 at the N-terminus of TRPM4b is critical for 14-3-3γ binding by showing GST pull-down and co-immunoprecipitation. Heterologous overexpression of 14-3-3γ in HEK293T cells increased TRPM4b expression on the plasma membrane which was measured by whole-cell recordings and cell surface biotinylation experiment. Surface expression of TRPM4b was greatly reduced by short hairpin RNA (shRNA) against 14-3-3γ. Next, endogenous TRPM4b-mediated currents were electrophysiologically characterized by application of glutamate and 9-phenanthrol, a TRPM4b specific antagonist in HT-22 cells which originated from mouse hippocampal neurons. Glutamate-induced TRPM4b currents were significantly attenuated by shRNAs against 14-3-3γ or TRPM4b in these cells. Finally, glutamate-induced cell death was greatly prevented by treatment of 9-phenanthrol or 14-3-3γ shRNA.

**Conclusion:**

These results showed that the cell surface expression of TRPM4 channels is mediated by 14-3-3γ binding, and the specific inhibition of this trafficking process can be a potential therapeutic target for glutamate-induced neuronal cell death.

## Background

The calcium ion (Ca^2+^) is the most abundant signaling molecule in regulating physiological and cellular functions [[1]]. Therefore, it is natural that Ca^2+^-permeable membrane proteins have been vigorously studied to understand their dynamic actions: voltage-gated Ca^2+^ channels, Ca^2+^-permeable TRP (Transient Receptor Potential) channels, ligand-gated Ca^2+^-permeable receptors (e.g., ionotrophic glutamate receptors, purinergic receptors and nicotinic acetylcholine receptors), and intracellular receptors such as ryanodine and IP_3_ receptors [[2]-[5]]. In addition, Ca^2+^-activated K^+^ channels (BK, IK, and SK), and Ca^2+^-activated Cl^−^ channels (Bestrophins and Anoctamins) are getting more attentions recently [[6],[7]].

Although Ca^2+^-activated Na^+^ channels have yet to be found, Ca^2+^-permeable non-selective cation channels are numerous (e.g., NMDA receptors and TRPC channels). In contrast, Ca^2+^-impermeable non-selective cation channels are only a few: AMPA receptors (except for AMPA receptors with calcium permeable unedited GluR2 subunit or without GluR2 subunit) and TRPM4 and TRPM5 [[8]-[11]]. TRPM4 and TRPM5 have been identified as Ca^2+^-activated, Ca^2+^-impermeable monovalent cation channels whose activities have been first reported more than three decades ago [[12]-[16]]. They conduct Na^+^ and K^+^ according to the concentration gradient of individual ion when activated by membrane depolarization and an increase in intracellular calcium ([Ca^2+^]_i_) [[14]-[17]]. They belong to a subgroup (melastatin-related TRPM) of TRP channels with six transmembrane domains which forms a functional channel as a tetramer [[18],[19]].

TRPM4 channels are expressed in various tissues including brain [[20]-[22]]. Mutations in TRPM4 gene and their consequential dysfunction have been linked to cardiac diseases [[23]-[25]]. The increased level of TRPM4 channels has been reported in vascular endothelium following hypoxia/ischemia, in the endothelial cells of capillary vessels following spinal cord injury [[26],[27]]. In the brain, expression and/or channel activities of TRPM4 have been detected in hippocampus, cerebellar Purkinje cells, preBӧtzinger complex in the brainstem, magnocellular cells in supraoptic and paraventricular nuclei, and substantia nigra pars compacta [[28]-[32]]. Recently, TRPM4 has been shown to be important in neuronal cell death of experimental autoimmune encephalomyelitis and human multiple sclerosis tissues [[31]]. Therefore, understanding trafficking mechanism of TRPM4 channels to the plasma membrane may open the therapeutic window to intervene the underlying TRPM4-related diseases.

## Results and discussions

### Identification of 14-3-3γ as a binding partner of TRPM4b

**Figure 1:**
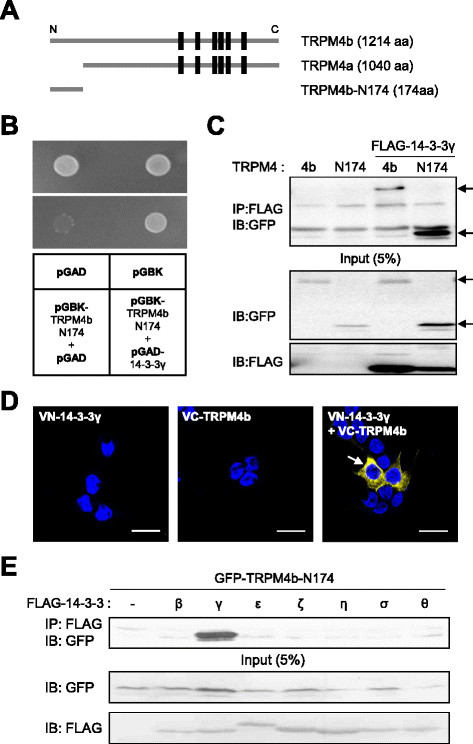
**TRPM4b associates with 14-3-3γ in heterologous systems. (A)** Diagram of the full length TRPM4b, a splice variant TRPM4a, and a short TRPM4b N-terminal fragment (TRPM4b-N174) used in this study. **(B)** Identification of 14-3-3γ as a direct binding partner of TRPM4b in the yeast two-hybrid system. **(C)** Co-IP experiment. GFP-TRPM4b (4b) or GFP-TRPM4b-N174 (N174) was transfected with or without FLAG-14-3-3γ in HEK293T cells and cell lysates were then immunoprecipitated using anti-FLAG antibody. Immunoprecipitates were examined by Western blotting using anti-GFP antibody (upper panel). Input represented 5% of cell lysates used in the Co-IP experiment (lower panel). **(D)** BiFC experiment with VN-14-3-3γ and VC-TRPM4b, where N- and C-terminal halves of Venus fluorescent protein were fused to 14-3-3γ and TRPM4b, respectively. Intense fluorescent signals were detected by the yellow color (arrow). Scale bar, 10 μm. **(E)** 14-3-3γ specifically binds to GFP-TRPM4b-N174 among seven 14-3-3 isoforms.

We previously reported that TRPM4 isoforms have differential expression on the plasma membrane: the full-length TRPM4b channels are highly localized on the plasma membrane, in contrast, TRPM4a lacking the N-terminal 174 amino acids is rarely present on the plasma membrane [[22]]. To identify the potential binding partner(s) for the surface expression of TRPM4b based on this observation, we made a bait construct of the intracellular N-terminal fragment (N174) of human TRPM4b for the conventional yeast two-hybrid (Y2H) screening (Figure 1A). Among the positive clones, we found human 14-3-3γ as a binding partner for TRPM4b (Figure 1B). They formed a positive yeast colony in Y2H under the non-permissive condition, while the empty vector did not. To examine the interaction between TRPM4b and 14-3-3γ in the mammalian system, we constructed expression vectors for FLAG-tagged 14-3-3γ (FLAG-14-3-3γ) and GFP-tagged TRPM4b (GFP-TRPM4b) or GFP-tagged N174, and co-expressed them in HEK293T cells. We then performed co-immunoprecipitation (Co-IP) on cell lysates with an anti-FLAG antibody and blotted with an anti-GFP antibody. The results showed that GFP-TRPM4b was strongly associated with FLAG-14-3-3γ (Figure 1C). The inverse experiment with anti-GFP antibody for Co-IP and anti-FLAG antibody for blotting showed the consistent result (see Additional file 1: Figure S1A). We then examined the interaction between TRPM4b and 14-3-3γ at the single-cell level to verify this interaction. We used the bimolecular fluorescence complementation (BiFC) technique, which allows visualization of two independent proteins in close spatial proximity [[33]]. We constructed TRPM4b and 14-3-3γ, whose N- and C- termini were fused to one complementary half of split Venus fluorescent protein, either N-terminal half (VN) or C-terminal half (VC), and then both were transfected into HEK293T cells (Figure 1D). Strong fluorescence was detected when the split Venus halves were on complementary positions (VC-TRPM4b and VN-14-3-3γ). The BiFC fluorescent signal was detected at the plasma membrane of the cells, shown in yellow (Figure 1D). In contrast, weak BiFC signal was detected from the cells transfected with VC-TRPM4a and VN-14-3-3γ (see Additional file 1: Figure S1B top panel). As a negative control, when TRPM4b or 14-3-3γ was expressed with only one half of split Venus, no fluorescence was detected. These results suggest that an association between TRPM4b and 14-3-3γ occurs in live mammalian cells. We further examined that other 14-3-3 isoforms could bind to N174 by using several FLAG-tagged 14-3-3 isoforms for Co-IP with anti-FLAG and anti-GFP antibodies. The result showed that binding of 14-3-3γ to TRPM4b is isoform-specific (Figure 1E). BiFC experiment using TRPM4b and 14-3-3σ produced the weak signal (see Additional file 1: Figure S1B low panel).

### Ser88 at the N-terminus of TRPM4b is critical for 14-3-3γ binding

**Figure 2:**
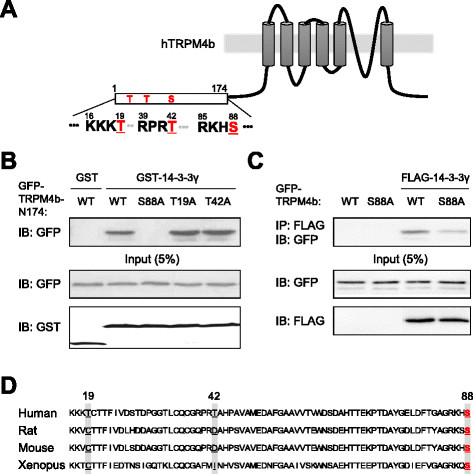
**Interaction of TRPM4b with 14-3-3γ may require a conserved serine phosphorylation residue. (A)** Schematic diagram depicting the membrane topology of hTRPM4b channels. Conserved phosphorylation residues (T19, T42, and S88) predicted for 14-3-3 binding were marked in the intracellular N-terminal domain (N174). These residues are highlighted in red. **(B)** GST-pulldown was performed on lysates of HEK293T cells co-expressing GST-14-3-3γ and GFP-TRPM4b-N174 or each mutant (S88A, T19A, and T42A). Cell lysates were immunoprecipitated using anti-GST antibody and immunoprecipitates were examined by Western blotting using anti-GFP or anti-GST antibody. Input represented 5% of cell lysates used. **(C)** Co-IP experiment was performed on lysates of HEK293T cells co-expressing FLAG-TRPM4b (WT) and its mutant (S88A). Cell lysates were immunoprecipitated using anti-FLAG antibody and immunoprecipitates were examined by Western blotting using anti-GFP or anti-FLAG antibody. Input represented 5% of cell lysates. **(D)** Sequence comparison of the region of conserved phosphorylation sites for 14-3-3 binding in hTRPM4b among human, rat, mouse and *Xenopus*. Only Ser88 was conserved over the species.

Next, we investigated which amino acid residues of TRPM4b are critical for 14-3-3γ binding. Through the Motif Scan database [http://scansite.mit.edu/motifscan_seq.phtml] three putative phosphorylation sites for 14-3-3γ binding were predicted on the N174 (Figure 2A). To identify the critical residue(s) for 14-3-3γ binding we replaced Thr19, Thr42 and Ser88 sites of GFP-TRPM4b-N174 to Ala individually (T19A, T42A, and S88A). From GST (Glutathione-S-transferase)-pull down with these mutants, we found that only S88A mutant proteins failed to interact with 14-3-3γ (Figure 2B). Co-IP experiment also revealed a drastic reduction in binding of S88A mutant of TRPM4b with 14-3-3γ (Figure 2C). In addition, a multiple sequence alignment shows that Serine 88 of TRPM4b is highly conserved in four vertebrate species (Figure 2D).

### Surface expression of TRPM4b is mediated by 14-3-3γ

**Figure 3:**
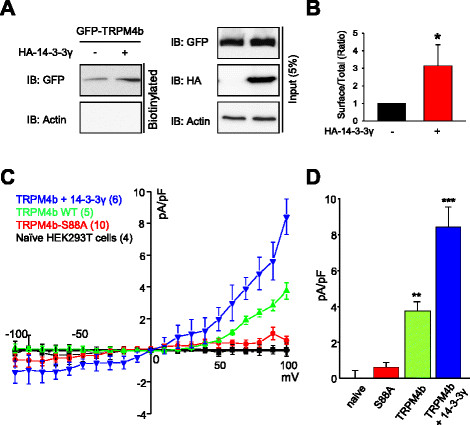
**The current density of TRPM4b channel is increased by 14-3-3γ co-expression. (A)** Surface biotinylation experiment was performed from HEK293T cells expressing GFP-TRPM4b with or without HA-14-3-3γ. Overexpression of 14-3-3γ markedly increased cell surface expression of TRPM4b. **(B)** Summary bar graph of TRPM4b level with or without 14-3-3γ co-expression was shown as a ratio of surface to total TRPM4b (n = 4). **(C)** The current densities were obtained from whole-cell currents of non-transfected HEK293T cells (black; n = 4), cells transfected with GFP-TRPM4b (green; n = 5), cells transfected with S88A TRPM4b mutant (red; n = 10) and cells co-transfected with GFP-TRPM4b and 14-3-3γ (blue; n = 6). Currents were activated by 1 μM ionomycin and the voltage-ramp (−100 mV to +100 mV). The current densities were calculated by subtracting the amplitude of initial currents from 1 μM ionomycin-activated currents and divided by cell capacitance. **(D)** Summary bar graph of TRPM4b currents with or without 14-3-3γ co-expression was plotted at +100 mV (** p < 0.01 and ***p < 0.001). All values are mean ± SEM.

Based on our result that TRPM4b binds to 14-3-3γ and on the information that 14-3-3 proteins are deeply involved in trafficking of membrane proteins [[34]], we hypothesized that 14-3-3γ increases the surface expression of TRPM4b channels. In cell surface biotinylation experiment, GFP-TRPM4b was markedly increased on the plasma membrane in the presence of HA-14-3-3γ (Hemagglutinin-tagged 14-3-3γ); however no significant changes in total cell lysate levels of TRPM4b were detected (Figure 3A and B).

Next, we measured TRPM4b-mediated whole-cell currents elicited by 1 μM ionomycin from the HEK293T cells overexpressing GFP-TRPM4b with or without mCherry-14-3-3γ using the voltage-ramp from −100 to +100 mV [[15]]. Currents were measured at 5 min after ionomycin application and ionomycin-activated currents were calculated by subtracting the initial currents from them (Figure 3C and D). The current density from cells overexpressing TRPM4b and 14-3-3γ were significantly larger than the ones from cells overexpressing TRPM4b alone. In addition, the currents from cells overexpressing TRPM4b-S88A mutants were negligible and similar to the ones from non-transfected cells. Alternatively, using the pipette solution including 30 μM [Ca^2+^]_i_ the current density from the cells overexpressing both TRPM4b and 14-3-3γ were more than two-folds larger than those from the cells overexpressing TRPM4b alone (see Additional file 2: Figures S2A and B).

### Deficiency of 14-3-3γ reduces the surface expression of TRPM4b in HEK293T cells

**Figure 4:**
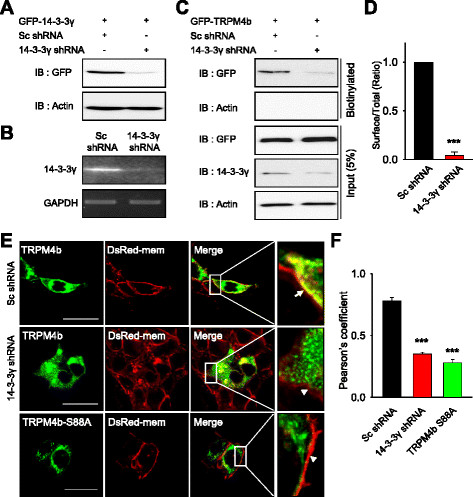
**The shRNA-mediated knockdown of 14-3-3γ reduced the surface expression of TRPM4b channels. (A)** 14-3-3γ shRNA eliminated GFP-14-3-3γ proteins on the Western blot from HEK293T cells overexpressing GFP-14-3-3γ (GFP, upper panel). Actin was used as a normalized control. **(B)** 14-3-3γ shRNA reduced the level of 14-3-3γ mRNA in HEK293T cells as examined by RT-PCR. GAPDH was used as an internal control. **(C)** Surface biotinylation experiment was performed from HEK293T cells expressing GFP-TRPM4b with Scrambled (Sc) or 14-3-3γ shRNA. The knockdown of 14-3-3γ markedly decreased cell surface expression of TRPM4b. **(D)** Summary bar graph of TRPM4b level with Sc or 14-3-3γ shRNAs co-expression was shown as a ratio of surface to total TRPM4b (n = 4). **(E)** GFP-TRPM4b was co-transfected with Sc shRNA (upper panel) or 14-3-3γ shRNA (middle panel) in HEK293T cells with DsRed-Mem, a plasma membrane marker. Intracellular localization of GFP-TRPM4b was visualized by confocal microscopy (left panels). GFP-TRPM4b-S88A was also shown in (bottom panel). Merged images were shown (right panel) and plasma membranes were indicated by an arrow and arrowheads in enlarged image. Scale bars, 10 μm. **(F)** The Pearson’s correlation coefficient for TRPM4b with 14-3-3γ shRNA or TRPM4b-S88A was significantly less than values obtained with Sc shRNA in HEK293T cells (P < 0.001). The Pearson’s correlation coefficient denoting covariance of fluorescence signals was calculated using Nikon A1 software.

To examine the effect of depletion of endogenous 14-3-3γ on the cell surface expression of overexpressed TRPM4b channels, we constructed a specific shRNA against 14-3-3γ and verified the efficiency of 14-3-3γ shRNA. There was little 14-3-3γ protein from the cells co-transfected with GFP-14-3-3γ cDNA and 14-3-3γ shRNA in contrast to the cells co-transfected with scrambled (Sc) shRNA by Western blot (Figure 4A). Endogenous 14-3-3γ mRNAs in HEK293T cells were also efficiently knock-downed in 14-3-3γ shRNA-transfected cells as shown in RT-PCR (Figure 4B). Next, we examined whether the surface expression of TRPM4b can be reduced by 14-3-3γ shRNA using cell surface biotinylation experiment. As expected, the surface expression of TRPM4b was markedly reduced (Figure 4C and D). We examined the co-localization with GFP-TRPM4b and DsRed-Mem, a plasma membrane marker to determine the effect of 14-3-3γ shRNA on the subcellular localization of TRPM4b (Figure 4E). GFP-TRPM4b channels were highly localized at the plasma membrane of HEK293T cells in the presence of Sc shRNA, as evidenced by a yellow color in the enlarged image (arrow in the top right panel), while they were less localized at the plasma membrane in the presence of 14-3-3γ shRNA (arrowhead in the middle right panel). As shown in Figures 2B and C, TRPM4b-S88A mutant, which 14-3-3γ interaction was greatly reduced, was also less localized on the plasma membrane (arrowhead in the bottom right panel). Reduced membrane localizations of TRPM4b with 14-3-3γ shRNA and TRPM4b-S88A were shown in a bar graph of Pearson’s correlation coefficient (P < 0.001; Figure 4F).

### Glutamate-induced activation of endogenous TRPM4b in neuronal cells from hippocampus

Recently, the deficiency of TRPM4 has been shown to be neuroprotective from glutamate-induced cell death in experimental autoimmune encephalomyelitis, a mouse model of human multiple sclerosis [[31]]. This report leads us to hypothesize that interference of TRPM4b function via blocking its trafficking to the plasma membrane with 14-3-3γ shRNA may have a protective effect from glutamate neurotoxicity. To test this hypothesis we first characterized TRPM4b-mediated currents in HT-22 cells, originated from mouse hippocampal neurons, which have been frequently used as an *in vitro* model for glutamate-induced neurotoxicity [[35]].

**Figure 5:**
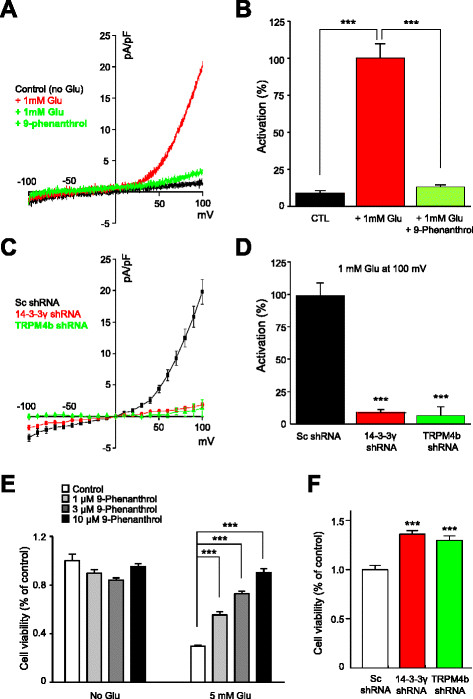
**Inhibition of TRPM4b channel activity increases cell survival from glutamate-mediated excitotoxicity in HT-22 cells. (A)** A representative whole-cell recording showed that the endogenous TRPM4b activation was induced by glutamate (1 mM) application and TRPM4b-activated currents were inhibited by 9-phenanthrol in HT-22 cells. **(B)** The summary bar graph showed the mean values of activation of TRPM4b by glutamate (1 mM) and its inhibition by 9-phenanthrol (100 μM) in HT-22 cells (P <0.001; n = 7). **(C)** Glutamate-induced TRPM4b currents in HT-22 cells was impaired with 14-3-3γ or TRPM4b shRNAs but not with Sc shRNA (n = 6). **(D)** The bar graph showed that the current amplitudes of TRPM4b channels transfected either with 14-3-3γ, TRPM4b or Sc shRNAs (P <0.001; n = 6). **(E)** The bar graph showed the results of MTT assay which glutamate-induced cell death was prevented by 9-phenanthrol dose-dependently (P <0.001; n = 24). **(F)** The bar graph showed that shRNA against 14-3-3γ or TRPM4b shRNAs significantly reduced glutamate-induced cell death in HT-22 cells (P <0.001; n = 24).

We measured whole-cell currents which were elicited by voltage-ramp pulses (400 ms) assessing from −100 mV to +100 mV from the holding potential of 0 mV. After obtaining steady-state responses (10–20 sweeps), glutamate (1 mM) was applied to the bath solution. Glutamate-induced currents were elicited within 3–4 minutes upon glutamate application and they reached near-maximal values within 10 minutes as originally shown [[16]] (see Additional file 3: Figure S3A). The glutamate-induced component had strong outward rectification and the reversal potential was close to 0 mV (Figure 5A and B). The current amplitude of glutamate-induced currents depicted at 100 mV was 9.87 ± 2.0 (mean ± SEM, n = 6) folds larger than the ones before glutamate application (Figure 5A and B). To verify whether the glutamate-induced currents were TRPM4-mediated, we used 9-phenanthrol, a selective TRPM4-specific antagonist [[36]]. 9-phenanthrol was applied to the bath solution 10 min after glutamate application when near-maximal glutamate-induced currents were obtained. Within 2 minutes of application of 9-phenanthrol (100 μM), the glutamate-induced currents were inhibited by 82.8 ± 2.9%, which is similar to the reported percentage of TRPM4 inhibition by 9-phenanthrol [[37]]. In addition, in the presence of 9-phenanthrol, glutamate failed to activate TRPM4b-mediated currents in HT-22 cells (see Additional file 3: Figure S3B).

We also examined the endogenous expression of TRPM4b in HT-22 cells by quantitative RT-PCR. As shown in Additional file 3: Figure S3C, TRPM4b expression in HT-22 cells was comparable to its expression in cultured hippocampal neurons. To rule out the possibility of glutamate-induced current activation through other than TRPM4b channels, we designed specific shRNAs against TRPM4b and found two functional shRNA constructs (see Additional file 3: Figure S3D). Glutamate-elicited currents was indeed failed to be activated in HT-22 cells transfected with a specific TRPM4b shRNA (Figure 5C and D). In summary, this data clearly showed that HT-22 cells have functional TRPM4b channels which can be elicited by glutamate.

Now, to examine the efficient interference of TRPM4b trafficking with 14-3-3γ shRNA, we measured glutamate-induced TRPM4b currents in HT-22 cells transfected with either 14-3-3γ shRNA or Sc shRNA. In contrast to the current amplitude of glutamate-induced TRPM4b channels in the cells transfected with Sc shRNA, the one in the cells transfected with 14-3-3γ shRNA was drastically reduced even 20 min after glutamate application (P < 0.001; Figure 5C and D).

Lastly, we asked whether inhibition of activity and trafficking of TRPM4b may prevent the neuronal cells from glutamate-induced cell death. First, we tested the effect of 9-phenanthrol on the glutamate-induced cell death using the MTT assay since 9-phenanthrol blocked TRPM4b channels effectively in whole-cell recordings. The great number of HT-22 cells was killed after glutamate (5 mM) treatment (69.57 ± 0.92% (n = 24); Figure 5E). In comparison, the cell death was dramatically reduced by 9-phenanthrol in a dose-dependent manner and no significant difference in control cells (without glutamate treatment) was detected (Figure 5E). We also examined the effect of 14-3-3γ shRNA on the cell viability in glutamate-treated HT-22 cells. Compared to the HT-22 cells transfected with Sc shRNA, knockdowns of 14-3-3γ and TRPM4b increased the cell survival (136.1 ± 3.6% (n = 24) and 129.8 ± 4.6% (n = 6), respectively; Figure 5F).

### 14-3-3γ-mediated TRPM4b trafficking mechanism provides a potential therapeutic target

14-3-3 proteins are one of most important ‘hub’ proteins involved in diverse cellular processes in all eukaryotes [[34]]. Their isoforms are broadly expressed in nervous systems and involved in many physiological conditions (such as hippocampal long-term potentiation) as well as neurodegenerative and neuropsychiatric diseases [[38]-[41]]. 14-3-3 proteins bind to a lot of membrane proteins including ion channels such as TASK-1 and TASK-3, TRESK, K_ATP_ channels, hERG/I_Kr_ channels, Na_V_1.5, Ca_V_2.2, GABA_B_ receptors, neuronal nicotinic α4β2 receptors, NMDA receptor subunit NR2C, kainate receptor subunit GluK2 and GluK5 [[42]-[53]]. Among isoforms, 14-3-3γ has been shown to interact with a large-conductance Ca^2+^-activated K^+^ (BK) channels and increases their surface expression as shown in this study [[54]]. In general, 14-3-3 proteins bind to (mostly) phosphorylated target proteins at the specific site(s) and function as a dimer [[34]]. A previous study showed that PKCδ activation increased the surface expression of TRPM4 channels in vascular smooth muscle cells [[55]]; however, two putative PKC phosphorylation sites are localized at the C-terminus of TRPM4 channels [[56]]. Since 14-3-3γ binds to the N-terminus of TRPM4b in this study, kinase(s) other than PKC may be involved in 14-3-3γ-mediated trafficking of TRPM4b channels.

A recent report showed that the glutamate-induced neuronal cell death was absent in TRPM4 null mice [[31]] and our results also clearly showed that inhibition of TRPM4b function either by a specific inhibitor or interfering trafficking of TRPM4b channels to the plasma membrane via 14-3-3γ shRNA blocked glutamate-induced cell death (Figure 5). Therefore, TRPM4b channels can be new therapeutic targets. R18 and difopein (dimeric fourteen-three-three peptide inhibitor), peptide inhibitors against 14-3-3 proteins, are available, however, specific inhibitors against each 14-3-3 isoforms have not been developed for the therapeutic purpose [[57]]. Instead, a gene therapy using shRNA against 14-3-3γ in a cell type-specific manner could be one promising way to ameliorate devastating neurological diseases. Especially, 14-3-3γ has been deeply involved in neurological diseases; inflammatory joint diseases [[58]], Alzheimer’s diseases [[59]], Parkinson’s disease [[60]], multiple sclerosis [[31]], and Creutzfeldt-Jakob disease [[61],[62]]. Whereas TRPM4b channels have not been studied in these diseases, 14-3-3γ-mediated TRPM4b trafficking might be involved in the neuronal cell death in these diseases.

## Conclusions

TRPM4b channels are Ca^2+^-activated, Ca^2+^-impermeable monovalent cation channels which are expressed in various neuronal tissues and involved in diverse physiological conditions. We have identified 14-3-3γ as a trafficking molecule for TRPM4b and Ser88 residue at the N-terminus of TRPM4b is critical for 14-3-3γ binding. Heterologous overexpression of 14-3-3γ showed the increased TRPM4b expression on the plasma membrane, in contrast, the surface expression of TRPM4b was greatly reduced by the knockdown of endogenous 14-3-3γ. Glutamate-induced TRPM4b currents and glutamate-induced neurotoxicity in HT-22 cells were attenuated by a TRPM4b specific antagonist and 14-3-3γ shRNA. Taken together, these results suggested that specific inhibition of 14-3-3γ-mediated trafficking of TRPM4b channels provide a new therapeutic target against neuronal cell death.

## Methods

### Chemicals

Ionomycin, 9-phenanthrol, 3-(4,5-dimethyl-2-thiazolyl)-2,5-diphenyl-2H-tetrazolium bromide (MTT) and all other chemicals were purchased from Sigma Aldrich.

### Plasmid and shRNA construction

The cDNA encoding for full-length human TRPM4b (Genebank accession no. NM_017636), mouse TRPM4b (Genebank accession no. NM_175130), and human 14-3-3γ (Genebank accession no. NM_012479) were obtained by the PCR-based gateway cloning method (Invitrogen). The cDNAs of 14-3-3 isoforms were kindly provided by Dr. Dukryong Kim (Gyeoungsang National University). TRPM4b-N174 was generated from the full-length cDNA, and its phosphorylation mutants (T19A, T42A and S88A) were made by EZchange site directed mutagenesis kit (Enzynomics). All constructs were cloned into several vectors, including pDEST-EGFP-C, pDEST-mCherry-C, pDEST-FLAG-C, pDEST-HA-C by gateway cloning. The target sequence of shRNAs as follows: human 14-3-3γ 5’-ACGAGGACTCCTACAAGGAC-3’, mouse 14-3-3γ 5’-GGACAACTACCTGATCAAGAA-3’, mouse TRPM4b 5’-GCTGGATCCCTAAGATCTTCA-3’.

### Yeast two-hybrid screening

The N-termini of human TRPM4b (N174) was ligated into pGBKT7 encoding for the GAL4 DNA binding domain (BD) and human cDNA library was cloned into pGADT7 encoding for the activation domain (AD). BD/TRPM4b-N174 and AD/cDNA library were co-transformed into the yeast strain AH109 in order to find protein-protein interactions with TRPM4b-N174. AH109 was unable to synthesize histidine but AD/14-3-3γ was found to grow on His minimal medium by permitting histidine biosynthesis.

### Cell culture and transfection

HEK293T and HT-22 cells were purchased from the Korean Cell Line Bank (Seoul National University) and cultured in DMEM (Invitrogen) supplemented with 10% fetal bovine serum (Invitrogen), 100 units per ml penicillin-streptomycin (Invitrogen) at 37°C in a humidified atmosphere of 95% O_2_ and 5% CO_2_. Transfection of expression vectors was performed with Lipofectamine 2000 (Invitrogen) according to the manufacturer’s protocol.

### Co-immunoprecipitation and immunoblotting

For co-immunoprecipitation, whole-cell lysates were mixed at 4°C with 1 μg/ml anti-FLAG (M2, sigma) antibodies in lysis buffer (in mM, 50 Tris–HCl (pH 7.4), 150 NaCl, 5 EDTA, 1 PMSF and 1% NP-40) containing a protease-inhibitor cocktail (Roche). Immune complexes were incubated by binding to mixed protein A/G PLUS-Agarose (Santa Cruz Biotechnology) for 1 hr, and washed four times with lysis buffer. For immunoblotting, Proteins were separated by 10% SDS-PAGE gel and blotted onto PVDF membranes. The blots were blocked with 5% skim-milk in TBST at room temperature for 20 min and incubated for overnight at 4°C with anti-GFP antibody (1:500, Santa Cruz Biotechnology), anti-FLAG (1:1000, Sigma) or anti-HA antibody (1:500, Roche). Blots were then washed and incubated with horse-radish peroxidase conjugated goat anti-mouse IgG, followed by washing and detection of immunoreactivity with the enhanced chemiluminescence (Amersham Biosciences).

### GST pull-down experiment

To produce GST-14-3-3γ (14-3-3γ fused with Glutathione-S-transferase) construct, the full-length human 14-3-3γ was cloned into the pDEST15 vector (Invitrogen) via the gateway cloning system. GST-14-3-3γ proteins were expressed in bacteria and purified according to the manufacturer’s protocol (Amersham Biosciences). Cell lysates obtained from wildtype or mutants of TRPM4b-N174 transfected cells were incubated with GST-14-3-3γ purified from bacteria for 2 hrs, and then incubated with glutathione-Sepharose 4B beads (Amersham Biosciences). After 2 hrs incubation at 4°C, the beads were washed four times with ice cold lysis buffer. Bound proteins were eluted with SDS sample buffer, separated on 10% SDS–PAGE gels, and analyzed by Western blot.

### Cell Surface biotinylation

For surface biotinylation, the cell membrane proteins of GFP-TRPM4b transfected HEK293T cells were biotinylated with PBS containing Sulfo-NHS-SS-biotin (Pierce) at 4°C for 30 min. After biotinylation, cells were washed with quenching buffer (100 mM glycine in PBS) to remove excess biotin and washed three times with PBS. The cells were then lysed and incubated with high capacity NeutrAvidin-Agarose Resin (Thermo Science). After three washes with lysis buffer, bound proteins are eluted by SDS sample buffer and subjected to Western blot analysis.

### *Bimolecular fluorescence complementation* (*BIFC) experiment*

TRPM4b and 14-3-3γ were cloned into bimolecular fluorescence complement (pBiFC)-VN173 and pBIFC-VC173 vectors. HEK293T cells were co-transfected with cloned BiFC vectors and transfected with DsRed-Mem for the detection of plasma membrane. These cells were fixed with 4% paraformaldehyde for 20 min at room temperature and mounted with Dako fluorescence mounting medium. Venus fluorescence signals were observed by confocal microscopy (Nikon A1).

### Image analysis

Cell surface expression of TRPM4b was determined using a confocal microscope. HEK293T cells were plated onto glass coverslips and co-transfected with GFP-TRPM4b and DsRed-Mem. The cells were cultured for additional 24 hrs, washed twice with PBS, and directly observed under a confocal microscope. Pearson’s correlation coefficients were calculated from the co-localization signals at the edge region of cells using the Nikon A1 confocal software.

### Electrophysiology

The standard solution for bath and pipette contained, in mM: 1 MgCl_2_, 5.5 glucose, and 10 HEPES. Na-gluconate (bath, 145 and pipette, 120) was used. Pipette solution contained 10 mM EGTA and pH was adjusted with NaOH to 7.2, while bath solution contained 2 mM CaCl_2_ and adjusted to pH 7.35. K^+^ was not included in either recording or pipette solution to eliminate the Ca^2+^-activated K^+^ channels which might be endogenously expressed in either HT293T or HT-22 cells. In a few initial experiments, tetraethylammonium (TEA, 2 mM) was added to the bath solution, however, it did not show any difference from the data with no TEA. Therefore, the data with and without TEA were pooled. Patch pipettes were made from borosilicate glass capillaries (Warner Instruments, Inc.). Whole-cell currents were recorded using a patch clamp amplifier (Axopatch 200B, Axon Instrument, Inc.). The current–voltage relations were measured by applying ramp pulses (from – 100 mV to +100 mV during 400 ms) from a holding potential of 0 mV at room temperature. The sampling interval was 200 μs and the filter setting was 2 KHz. Currents were analyzed with Clampfit software (Axon instruments, Inc.). Ionomycin (1 μM) was used to activate TRPM4b channels overexpressed in HEK293T cells. The current densities were calculated by subtracting the amplitude of initial currents from activated TRPM4b currents and divided by cell capacitance. A TRPM4b inhibitor, 9-phenanthrol (100 μM) was applied when glutamate-induced TRPM4b currents reached to near maximum values (usually 10 – 20 minutes after glutamate (1 mM) application) to measure the inhibition in HT-22 cells.

### Real-time quantitative RT-PCR (qRT-PCR)

Total RNA was isolated from HT-22 cells and primary cultured hippocampal neurons using a RNA purification kit (GeneAll) according to the manufacturer’s instruction. The cDNA was synthesized from 1 μg of total RNA and reverse transcription was performed with SuperScript® VILO™ cDNA Synthesis Kit (Invitrogen). The qRT-PCR was done with a SensiFAST™ Probe Hi-ROX Kit (Bioline) using the TaqMan probe Mm.PT.58.8488376 (IDT). GAPDH was used as an endogenous control. The 2^-ΔΔCt^ method was used to calculate fold changes in gene expression [[63]].

### MTT cell viability experiment

Cell viability of HT-22 cells was assessed by MTT assay [[64]]. 9-phenanthrol (1, 3 and10 μM) was applied 30 min before incubating in glutamate (5 mM) for 18 hr. After treatment, MTT reagent (5 mg/ml in PBS) was added to the culture wells, incubated for 3 hr, and the samples were lysed with Dimethyl Sulfoxide (DMSO). MTT is reductively converted into a violet formazan derivative, which is quantified photometrically at 570 nm in a well plate reading system (Infinite M200 pro). The mean values from the readings of triplicate wells were taken as one value. The optical density value for the control cultures was considered as 100% viability (control) and viability in other samples is expressed as a percentage of viability in the control culture.

### Statistical analysis

Numerical data are presented as means ± SEM. Error bars in graphs denote the standard error of the mean. The statistical significance of data was assessed by unpaired or paired Student’s *t*-test. The significance level is displayed as asterisks (*P < 0.05 or **P < 0.01, ***p < 0.001).

## Abbreviations

CHPG: [(RS)-chloro-5-hydroxyphenylglycine]

GFP: (Green Fluorescent Protein)

MPEP: (2-methyl-6-(phenylethynyl)-pyridine)

## Competing interests

Authors declare no conflict of interests.

## Authors’ contribution

Drs. C-HC and EK have planned and performed the experiment and discussed the results with Drs. EMH, S-GH and J-YP and written the manuscript together. Dr. OY and Y-SL contributed to the study by performing some experiments (electrophysiology and biotinylation respectively). All authors read and approved the final manuscript.

## Additional files

## Supplementary Material

Additional file 1: Figure S1. **(A)** Inverse Co-IP experiment. GFP-TRPM4b (4b) or GFP-TRPM4b-N174 (N174) was transfected with or without FLAG-14-3-3γ in HEK293T cells and cell lysates were then immunoprecipitated using anti-GFP antibody. Immunoprecipitates were examined by Western blotting using anti-FLAG antibody (upper panel). Input represented 5% of cell lysates used in the Co-IP experiment (lower panel). **(B)** BiFC experiment with VC-TRPM4a with and without VN-14-3-3γ (top panel) and VN-14-3-3σ with and without VC-TRPM4b were shown (bottom panel). Weak Venus signals were detected by the yellow color (arrow) compared to the Figure 1D. Scale bar, 10 μm. Click here for file

Additional file 2: Figure S2. **(A)** The current densities were obtained from whole-cell currents of non-transfected HEK293T cells (black; n = 7), cells transfected with GFP-TRPM4b (green; n = 7), and cells co-transfected with GFP-TRPM4b and 14-3-3γ (red; n = 9). Currents were activated by 30 μM [Ca ^2+^]_i_ and the voltage-ramp (−100 mV to +100 mV). These overexpressed TRPM4b currents showed linear I-V relationships as previously shown when high [Ca^2+^]_i_ was used [[15]]. **(B)** Summary bar graph of TRPM4b currents with or without 14-3-3γ co-expression was plotted at −100 mV and +100 mV (***p < 0.001). All values are mean ± SEM. Click here for file

Additional file 3: Figure S3. **(A)** Activation time-course of TRPM4b-mediated currents elicited by glutamate (1 mM) application in HT-22 cells (n = 6). **(B)** A representative trace of whole cell recording showed that pre-incubation (5–15 min) of 9-phenanthrol (100 μM) failed to activate endogenous TRPM4b-mediated currents elicited by 10 min application of glutamate (1 mM) in HT-22 cells (n = 4; 0.292 ± 0.139 pA/pF increased at +100 mV). Note that raw traces were activated by voltage ramp (−100 to +100 mV) before (black) and 10 min after (red) glutamate application were overlapped. **(C)** Summary bar graph of qRT-PCR of TRPM4b in HT-22 cells. The level of endogenous TRPM4b mRNA in HT-22 cells was comparable to the one in mouse primary cultured hippocampal neurons. **(D)** Validation of mouse TRPM4b shRNA constructs. HEK293T cells were co-transfected with GFP-TRPM4b and TRPM4b shRNA1 or shRNA2 and their knockdown efficiency was evaluated by Western blot using anti-GFP antibody against GFP-TRPM4b. Click here for file
