# Supplementary material for: Depletion of 14-3-3γ reduces the surface expression of Transient Receptor Potential Melastatin 4b (TRPM4b) Channels and attenuates TRPM4b-mediated glutamate-induced neuronal cell death
Source: Mol Brain. 2014 Jul 22;7:52. doi: 10.1186/s13041-014-0052-3 (PMC4115172; doi:10.1186/s13041-014-0052-3)
Supplement: Additional file 1: Figure S1. — (A) Inverse Co-IP experiment. GFP-TRPM4b (4b) or GFP-TRPM4b-N174 (N174) was transfected with or without FLAG-14-3-3γ in HEK293T cells and cell lysates were then immunoprecipitated using anti-GFP antibody. Immunoprecipitates were examined by Western blotting using anti-FLAG antibody (upper panel). Input represented 5% of cell lysates used in the Co-IP experiment (lower panel). (B) BiFC experiment with VC-TRPM4a with and without VN-14-3-3γ (top panel) and VN-14-3-3σ with and without VC-TRPM4b were shown (bottom panel). Weak Venus signals were detected by the yellow color (arrow) compared to the Figure 1D. Scale bar, 10 μm. [file s13041-014-0052-3-S1.pdf]

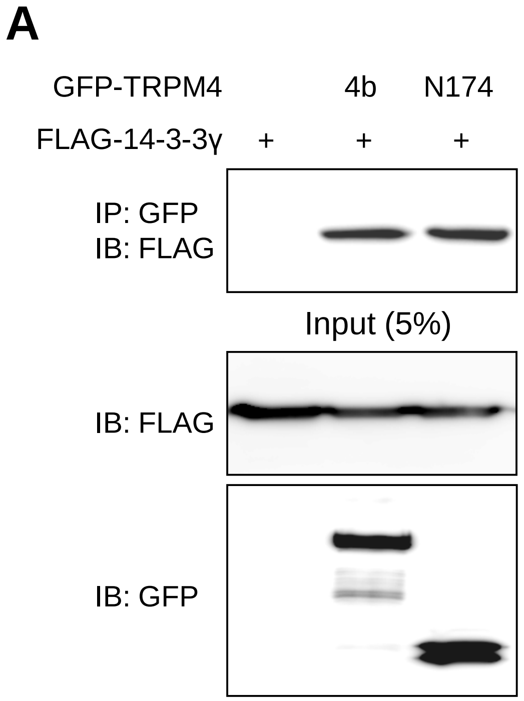

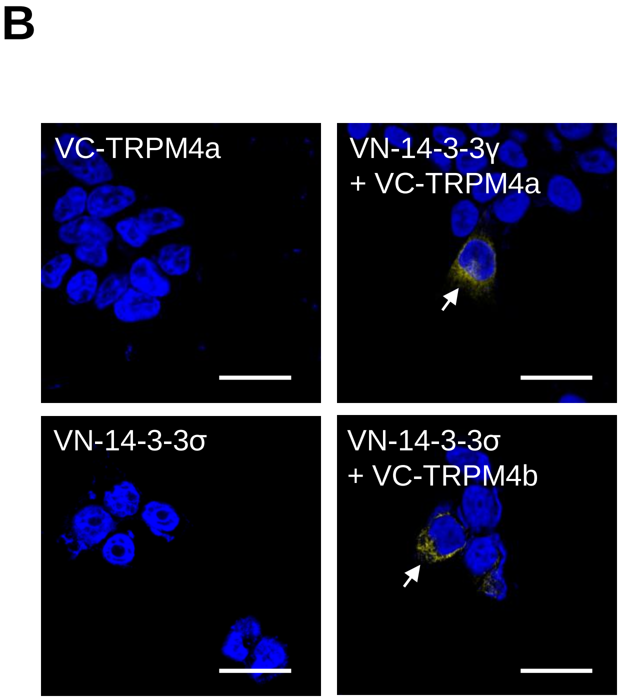

Supplementary Figure 1. (A) Inverse Co-IP experiment. GFP-TRPM4b (4b) or GFP-TRPM4b-N174 (N174) was transfected with or without FLAG-14-3-3γ in HEK293T cells and cell lysates were then immunoprecipitated using anti-GFP antibody. Immunoprecipitates were examined by Western blotting using anti-FLAG antibody (upper panel). Input represented 5% of cell lysates used in the Co-IP experiment (lower panel). **(B)** BiFC experiment with VC-TRPM4a with and without VN-14-3-3γ (top panel) and VN-14-3-3σ with and without VC-TRPM4b were shown (bottom panel). Weak Venus signals were detected by the yellow color (arrow) compared to the Fig. 1D. Scale bar, 10 μm.
