# Supplementary material for: Depletion of 14-3-3γ reduces the surface expression of Transient Receptor Potential Melastatin 4b (TRPM4b) Channels and attenuates TRPM4b-mediated glutamate-induced neuronal cell death
Source: Mol Brain. 2014 Jul 22;7:52. doi: 10.1186/s13041-014-0052-3 (PMC4115172; doi:10.1186/s13041-014-0052-3)
Supplement: Additional file 2: Figure S2. — (A) The current densities were obtained from whole-cell currents of non-transfected HEK293T cells (black; n = 7), cells transfected with GFP-TRPM4b (green; n = 7), and cells co-transfected with GFP-TRPM4b and 14-3-3γ (red; n = 9). Currents were activated by 30 μM [Ca 2+]i and the voltage-ramp (−100 mV to +100 mV). These overexpressed TRPM4b currents showed linear I-V relationships as previously shown when high [Ca2+]i was used [[15]]. (B) Summary bar graph of TRPM4b currents with or without 14-3-3γ co-expression was plotted at −100 mV and +100 mV (***p < 0.001). All values are mean ± SEM. [file s13041-014-0052-3-S2.pdf]

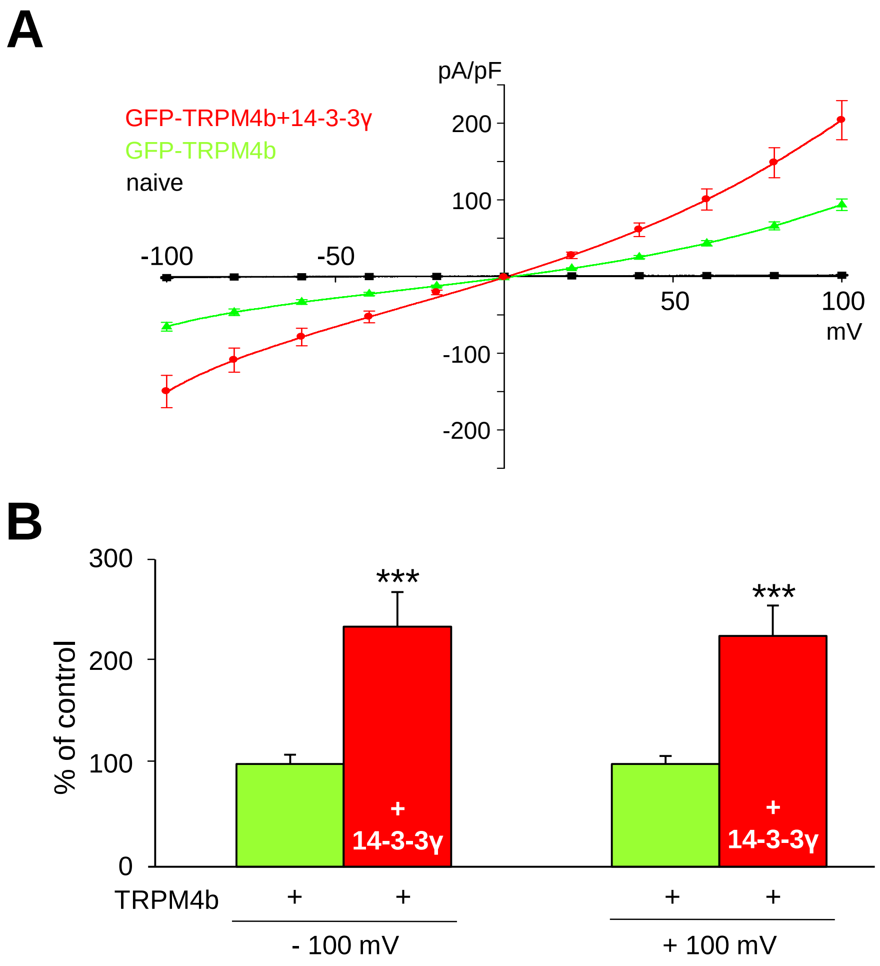

Supplementary Figure 2. (A) Whole-cell currents of non-transfected HEK293T cells (black; n=7), cells transfected with GFP-TRPM4b (green; n=7), and cells co-transfected with GFP-TRPM4b and 14-3-3γ (red; n=9). Currents were activated by 30 μM $[Ca^{2+}]_i$ and the voltage-ramp (-100 mV to 100 mV). **(B)** Summary bar graph of TRPM4b currents with or without 14-3-3γ co-expression was plotted at -100 mV and +100 mV (***p<0.001). All values are mean ± SEM.
