# Supplementary material for: Depletion of 14-3-3γ reduces the surface expression of Transient Receptor Potential Melastatin 4b (TRPM4b) Channels and attenuates TRPM4b-mediated glutamate-induced neuronal cell death
Source: Mol Brain. 2014 Jul 22;7:52. doi: 10.1186/s13041-014-0052-3 (PMC4115172; doi:10.1186/s13041-014-0052-3)
Supplement: Additional file 3: Figure S3. — (A) Activation time-course of TRPM4b-mediated currents elicited by glutamate (1 mM) application in HT-22 cells (n = 6). (B) A representative trace of whole cell recording showed that pre-incubation (5–15 min) of 9-phenanthrol (100 μM) failed to activate endogenous TRPM4b-mediated currents elicited by 10 min application of glutamate (1 mM) in HT-22 cells (n = 4; 0.292 ± 0.139 pA/pF increased at +100 mV). Note that raw traces were activated by voltage ramp (−100 to +100 mV) before (black) and 10 min after (red) glutamate application were overlapped. (C) Summary bar graph of qRT-PCR of TRPM4b in HT-22 cells. The level of endogenous TRPM4b mRNA in HT-22 cells was comparable to the one in mouse primary cultured hippocampal neurons. (D) Validation of mouse TRPM4b shRNA constructs. HEK293T cells were co-transfected with GFP-TRPM4b and TRPM4b shRNA1 or shRNA2 and their knockdown efficiency was evaluated by Western blot using anti-GFP antibody against GFP-TRPM4b. [file s13041-014-0052-3-S3.pdf]

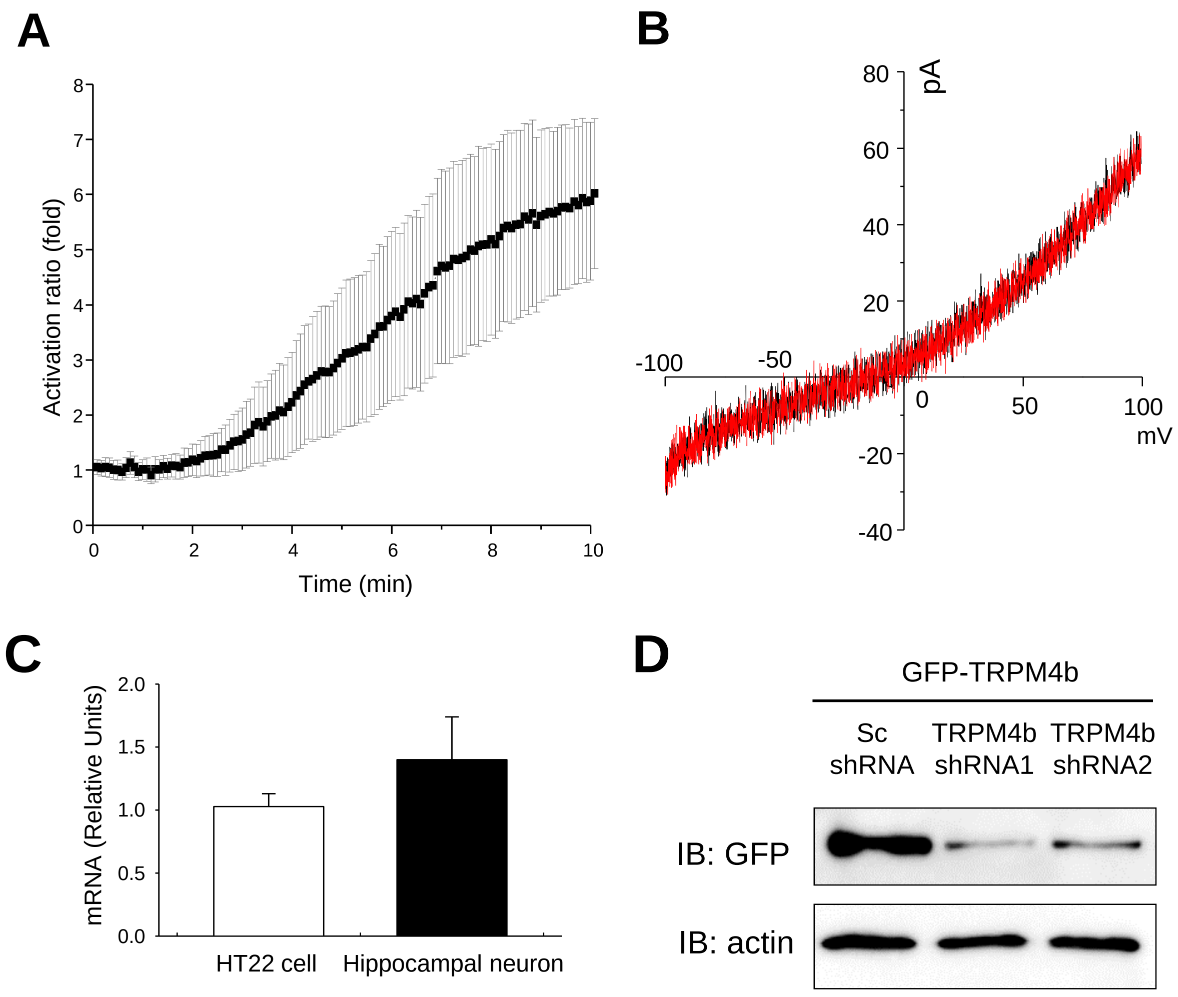

Supplementary Figure 3. (A) Activation time-course of TRPM4b-mediated currents elicited by 1 mM glutamate application in HT-22 cells (n=6). **(B)** A representative traces of whole cell recording showed that pre-incubation (5-15 min) of 9-phenantrol (100 μM) failed to activate endogenous TRPM4b-mediated currents elicited by 10 min application of glutamate (1 mM) in HT-22 cells (n=4; 0.292 ± 0.139 pA/pF increased). Note that raw traces were activated by voltage ramp (-100 to +100 mV) before (black) and 10 min after (red) 1 mM glutamate application were overlapped. **(C)** Summary bar graph of qRT-PCR of TRPM4b in HT-22 cells. The level of endogenous TRPM4b mRNA in HT-22 cells was comparable to the one in mouse primary cultured hippocampal neurons. **(D)** Validation of mouse TRPM4b shRNA constructs. HEK293T cells were co-transfected with GFP-mouse TRPM4b and mouse TRPM4b shRNA1 or shRNA2 and their knockdown efficiency was evaluated by Western blot using anti-GFP antibody against GFP-TRPM4b.
